# Blue light-emitting diode phototherapy presents *in vitro* efficacy against distinct *Leishmania* species and is therapeutic against tegumentary leishmaniasis in BALB/c mice

**DOI:** 10.3389/fimmu.2025.1554051

**Published:** 2025-05-05

**Authors:** Breno Luiz Pimenta, Daniela Pagliara Lage, Camila Simões de Freitas, Danniele Luciana Vale, Karolina Oliveira de Melo Falcão, Saulo Samuel Gonçalves Dias, Raquel Soares Bandeira Câmara, Isabela Amorim Gonçalves Pereira, Ana Luiza Silva, Lauro de Assis Duarte Júnior, Miguel Angel Chavez-Fumagalli, Rodrigo Fernando Bianchi, Eduardo Antonio Ferraz Coelho, André Talvani

**Affiliations:** ^1^ Programa de Pós-Graduação em Ciências da Saúde: Infectologia e Medicina Tropical, Faculdade de Medicina, Universidade Federal de Minas Gerais, Belo Horizonte, Brazil; ^2^ Laboratório de Imunobiologia da Inflamação, Departamento de Ciências Biológicas, Instituto de Ciências Exatas e Biológicas (ICEB), Universidade Federal de Ouro Preto, Ouro Preto, Brazil; ^3^ Departamento de Patologia Clínica, Colégio Técnico da Universidade Federal de Minas Gerais, Belo Horizonte, Brazil; ^4^ Laboratório de Polímeros e Propriedades Eletrônicas de Materiais, Instituto de Ciências Exatas e Biológicas (ICEB), Universidade Federal de Ouro Preto, Ouro Preto, Brazil; ^5^ Computational Biology and Chemistry Research Group, Vicerrectorado de Investigación, Universidad Católica de Santa María, Arequipa, Peru

**Keywords:** blue LED phototherapy, tegumentary leishmaniasis, treatment, amphotericin B, immune response, antileishmanial activity

## Abstract

The treatment of tegumentary leishmaniasis (TL) faces significant challenges, including drug toxicity, high costs, and the emergence of resistant strains. These limitations highlight the urgent need for novel antileishmanial agents and therapeutic strategies. This study evaluated blue light-emitting diode (LED) phototherapy as an alternative approach to inhibit *Leishmania* stationary promastigotes and treat infected mammalian models. *In vitro* assays using *Leishmania amazonensis*, *L. braziliensis*, and *L. infantum* demonstrated that blue LED significantly inhibited parasite growth during and after treatment, with inhibition levels comparable to those achieved with amphotericin B (AmpB). Treatment of infected macrophages with blue LED substantially reduced infection rates and amastigote recovery across all three parasite species. Ultrastructural analyses revealed the destruction of internal organelles and alterations to the surface membranes of all *Leishmania* species following blue LED exposure. In *in vivo* experiments, *L. amazonensis*-infected BALB/c mice were treated with AmpB, blue LED alone, combination of blue LED plus AmpB, or saline as a control. Animals treated with blue LED, particularly in combination with AmpB, exhibited significant reductions in parasite loads in infected tissues such as lesions, spleens, livers, and draining lymph nodes, as confirmed by limiting dilution assays and qPCR. Additionally, these treatments induced a robust antileishmanial Th1-type immune response, characterized by increased production of IFN-γ, IL-12, nitrite, and IgG2a antibodies. These findings suggest that blue LED phototherapy holds promise as a potential therapeutic strategy for TL and warrants further investigation in future studies.

## 1 Introduction

Leishmaniases are endemic diseases caused by more than 20 species of *Leishmania* parasites (1), which are distributed geographically across the world with different species able to cause distinct clinical manifestations ranging from self-limiting cutaneous lesions to life-threatening visceral disease (2). The infection outcome is mainly determined by factors related to parasite infectivity, vector biology, host immune responses and nutritional status (3). In this context, the main clinical forms of the disease are tegumentary leishmaniasis (TL) and visceral leishmaniasis (VL) (4); where TL is presented in the cutaneous, diffuse cutaneous and mucosal forms, being characterized by one or more lesions on the skin, which can cause destruction of the mucosa and cartilage leading to respiratory compromise in the patient (5). It can be caused by several parasite species, such as *Leishmania braziliensis, L. major, L. amazonensis, L. tropica*, and others. VL is the most serious form of the disease and can lead to death, causing symptoms such as fever, anorexia, diarrhea, hepatosplenomegaly, lymphadenopathy, and vascular problems (6).

Treatment for the disease is based on the use of pentavalent antimonials, free or liposomal amphotericin B (AmpB), pentamidine, miltefosine, among others; although they cause organ toxicity, are high in cost and/or lead to the emergence of resistant strains (7, 8). Given these limitations, there is a pressing need to develop safe and alternative approaches to conventional treatment. Besides new and promising synthetic molecules, delivery systems and therapeutic protocols, photodynamic therapy based on light interaction at a suitable wavelength with a photosensitive antileishmanial compound and oxygen can trigger a photochemical reaction and generate reactive oxygen species leading to cell death (9, 10). This strategy has been used as an adjuvant therapy to treat pulmonary, respiratory tract, neural, and urinary tract tumors, as well as vitreoretinal disease (11, 12). Different compounds, such as carbaporphyrin ketals, methylene blue, aminolevulinic acid, chloroaluminum phthalocyanine, and aluminum phthalocyanine tetrasulfonate have been employed as photosensitizers, because the investigation of new photosensitizers is important to improve the effectiveness of the therapy. Photodynamic therapy (PDT) has also emerged as an alternative for the treatment of TL, with studies on its effectiveness being conducted in humans and animal models (13, 14). In another promising experimental strategy, blue light-emitting diode (LED) phototherapy has also been proposed as a treatment for diseases (15, 16). Blue light is part of the natural light received from the sun, which has been shown to inactivate microorganisms such as bacteria and fungi (17, 18). The proposed action is similar to PDT, since it is based on the photoexcitation of endogenous porphyrins that increase the level of reactive oxygen species (19), causing damage to proteins and lipids in the microorganism’s membrane, thereby affecting intracellular transport (20). An additional effect of blue light is photo-immunomodulation because light, in general, affects the release of hormones and cytokines (21).

In a recent study, Ivanova et al. (22) proposed an innovative approach to controlling *Trypanosoma cruzi* infection using blue LED phototherapy. *In vitro* assays with axenic cultures of Y and CL strains of *T. cruzi* demonstrated a 50% reduction in epimastigote replication after five days of blue light exposure. *In vivo* experiments in C57BL/6 mice infected with the Y strain of *T. cruzi* revealed that blue LED phototherapy reduced trypomastigote levels in blood and cardiac tissue. Additionally, it lowered plasma levels of IL-6, TNF, and IL-10 (but not CCL2), suggesting a light-mediated parasite control. The authors claimed their study was the first to explore blue LED phototherapy for *T. cruzi* infection. However, no studies have evaluated this therapy for treating tegumentary leishmaniasis (TL).

So, the present study investigated the antileishmanial effects of blue LED phototherapy, both alone and in combination with amphotericin B (AmpB), against stationary-phase promastigotes of *L. infantum*, *L. braziliensis*, and *L. amazonensis*. The effects were also evaluated *in vitro* (axenic and peritoneal macrophage cultures) and *in vivo* (BALB/c mice). Additionally, the study assessed how chemotherapy and phototherapy influenced parasite ultrastructure and modulated the mammalian immune response.

## 2 Materials and methods

### 2.1 Ethical committee on animal research

The study was approved by the Ethical Committee on Animal Research (CEUA) of the Federal University of Minas Gerais (UFMG; Belo Horizonte, Minas Gerais, Brazil), with the protocol number 056/2022. Female BALB/c mice, aged 8 weeks, were obtained from the Center for Animal Facilities at the Institute of Biological Sciences of UFMG, and were kept under pathogen-free conditions.

### 2.2 *In vitro* activity against distinct Leishmania species

*L. amazonensis* (IFLA/BR/1967/PH-8), *L. braziliensis* (MHOM/BR/1975/M2904) and *L. infantum* (MHOM/BR/1970/BH46) were used. Stationary-phase promastigotes were cultured in complete Schneider’s medium (Sigma-Aldrich, USA), which was supplemented with 20% heat-inactivated fetal bovine serum (FBS; Sigma-Aldrich, USA), 20 mM L-glutamine, 200 U/mL penicillin, and 100 μg/mL streptomycin, at pH 7.4, at 24°C (23). All technical procedures were carried out under sterile conditions. Parasites (1 x 10^6^ cells/mL) were plated in complete Schneider’s medium with a final volume of 500 μL per well and submitted to blue LED phototherapy for 12 h per day over 5 days at 24°C. Daily, the emitted irradiance was checked with a radiometer to ensure consistency throughout the treatment. Some wells were untreated (background control) or treated with AmpB (Cat Y0001361 – European Pharmacopoeia. 0.1 μg/mL/well, 1 day at 24°C). Cell viability was assessed daily by counting viable parasites in a Neubauer chamber over five days (12h/day), as trypanosomatids exhibit partial resistance to phototherapy in the absence of mammalian immune cells (22).

### 2.3 Transmission electron microscopy

Parasite ultrastructural alterations were evaluated using transmission electron microscopy (TEM). Samples from the three *Leishmania* species, previously exposed to white and blue light for 5 days (12 hours/day), were fixed in a solution containing 2.5% glutaraldehyde, 4% paraformaldehyde, and 0.1 M phosphate buffer. The samples were then adhered to glass slides coated with 0.1% poly-L-lysine for 30 minutes at 37°C.

After fixation, the slides were washed twice with 0.1 M phosphate buffer, and postfixed in osmium tetroxide (OsO_4_) solution for 1 hour at room temperature, then washed again twice with 0.1 M phosphate buffer. The samples were gradually dehydrated through an ethanol series and analyzed using a Tecnai G2-12 FEI Spirit Biotwin 120 kV electron microscope at the Centro de Microscopia, UFMG, Belo Horizonte, Brazil.

### 2.4 Treatment using blue LED light phototherapy in Leishmania-infected macrophages

Treatment of infected murine peritoneal macrophages was performed by culturing cells (5 x 10^5^ per well) in complete RPMI 1640 medium (Sigma-Aldrich, USA), which was supplemented with 20% heat-inactivated fetal bovine serum (FBS; Sigma-Aldrich, USA), 20 mM L-glutamine, 200 U/mL penicillin, and 100 μg/mL streptomycin, for 2 h at 37°C with 5% (v/v) CO_2_. Next, non-adherent macrophages were removed by washing with a complete RPMI 1640 medium. *L. amazonensis*, *L. braziliensis* and *L. infantum* stationary-phase promastigotes (at a ratio of 10 parasites per macrophage) were added, and a new incubation was carried out for 24 h at 37°C with 5% CO_2_. Parasites were washed to remove non-adherent or non-phagocytized ones, and infected macrophages were treated with blue LED phototherapy or AmpB (1.0 μg/mL/well) for 24 or 48 h, at 37°C. Since parasites within peritoneal macrophages are subjected to immune pressure, the blue LED phototherapy protocol was adjusted accordingly. Unlike in axenic cultures, phototherapy was applied continuously for 24 or 48 h. After incubation, cells were stained using the panoptic method and, the percentage of infected macrophages and the number of intracellular amastigotes were determined by counting 200 cells per cover glass in triplicate under an optical microscope.

### 2.5 *In vivo* infection and treatment schedules

Two independent experiments were performed, and similar results were obtained. One representative experiment is shown in this work. BALB/c mice (n=4 per group) were infected subcutaneously on the dorsum with 10^6^ *L. amazonensis* stationary-phase promastigotes. After lesion development (at 50 to 60 days after infection), treatment was initiated with animals exposed to blue LED phototherapy for 12 h per day over 10 days, with the light positioned above the cage, directed onto the lesion. The phototherapy device was custom-built by an electrical engineer in our laboratory. It consists of a wooden box equipped with four blue LED lights positioned at the top, 30 cm above the polypropylene cages containing the mice. The intensity (7 μW/cm2) of blue light was verified daily with a portable radiometer, inside each cage, to guarantee uniform radiance for all animals. Each device was connected to an electrical power source, which accommodated four cages simultaneously. The animals remained in their usual confined environment within the cages, with food pellets provided inside to minimize any resistance to light exposure. Additionally, cooling fans were installed at the top of the device to prevent temperature increases inside the box, despite the room being temperature-controlled. A 24-hour surveillance camera was also placed on top of the device to monitor the animals’ environment and detect any potential disturbances.

Mice treated with AmpB (1.0 mg/kg/dose) or receiving saline were handled through intraperitoneal injections. The study design was as follows: (*i*) control group: uninfected mice received blue LED phototherapy for 12 h per day for 10 days; (*ii*) saline group: mice were infected and received 100 μL PBS 1x in 5 doses with one-day intervals; (*iii*) blue LED phototherapy group: mice were infected and treated with blue LED phototherapy for 12 h per day for 10 days; (*iv*) AmpB group: mice were infected and treated with AmpB (1 mg/kg/dose in 5 doses with one-day intervals); (*v*) blue LED phototherapy plus AmpB group: mice were infected and treated with blue LED phototherapy for 12 h per day for 10 days and AmpB (1 mg/kg/dose in 5 doses with one-day intervals). The development of nodules, metastasis and other clinical signs were monitored during and after treatment.

### 2.6 Cellular response and nitrite secretion

Cytokine production was evaluated in splenic cultures from infected and treated animals, as previously described (24). Briefly, splenic cell suspensions (1 x 10^6^ cells/well) were plated in duplicate in 24-well plates (Nunc) and incubated in complete RPMI 1640 (Sigma-Aldrich, USA). Cells were either unstimulated (medium) or stimulated with soluble *Leishmania* antigen - SLA (25 μg/mL) for 48 h at 37°C in 5% CO_2_. IFN-γ, IL-4, IL-10 and IL-12p70 levels were measured in the culture supernatant by capture ELISA using commercial kits (BD Pharmingen^®^, San Diego, CA, USA), according to manufacturer’s instructions. Nitrite production was assessed in the same culture supernatant, as previously described (25).

### 2.7 Antibody production evaluation

The humoral response was evaluated through the measurement of levels of total anti-SLA IgG and the ratio between IgG2a and IgG1 antibodies through an indirect ELISA, as previously described (24). After previous titration curves, SLA was added to the plates (1.0 μg per well), which were incubated for 16 h at 4°C. Then, 250 μL of a solution composed of 2% casein diluted in PBS-T (PBS 1x and 0.05% Tween 20) were added to the wells, and incubated for 1 h at 37°C. Plates were washed with PBS-T and mouse serum samples were added (1:50 diluted in PBS-T), and plates were again incubated for 1 h at 37°C. Plates were washed with PBS-T, and incubated with anti-mouse IgG total (goat anti-mouse IgG total secondary antibody, catalog SA1-31430, Invitrogen, USA), IgG1 (rat anti-mouse IgG1 secondary antibody, catalog SA1-35640, Invitrogen, USA) or IgG2a (rat anti-mouse IgG2a secondary antibody, catalog SA1-35646, Invitrogen, USA) antibodies. A new incubation was then performed for 1 h at 37°C, after which plates were washed with PBS-T and reactions were developed using H_2_O_2_, ortho-phenylenediamine, and a citrate-phosphate buffer pH 5.0 for 30 min in the dark. Next, they were stopped by adding 2N H_2_SO_4_ and optical density (OD) values were read in a spectrophotometer (Molecular Devices, Spectra Max Plus, Concord, Canada), at 492 nm.

### 2.8 Parasite load estimation

The parasite load was evaluated in the lesion, spleen, liver and draining lymph nodes (dLN) of infected and treated animals after therapy using a limiting dilution technique (26). Briefly, the skin lesions and organs were collected, weighed, and homogenized in a glass tissue grinder in sterile PBS 1x, and debris was removed by centrifugation at 150 x *g*. The suspension was plated in flat-bottom 96-well microplates (Nunc), and serial dilutions were made in complete Schneider’s medium from 10¹ to 10¹². Pipette tips were discarded after each dilution to prevent cross-contamination. Each sample was plated and incubated at 24°C and the presence of viable parasites was assessed seven days after culture initiation. Results were expressed as the negative log of the titer, which corresponded to the dilution in the last positive well, adjusted for tissue or organ weight. The *L. amazonensis* burden was also evaluated through a qPCR technique in skin lesions of infected mice, as previously described (27). Results were calculated by interpolation from a standard curve included in the same run, which was done in duplicate and expressed as the number of parasites per total DNA.

### 2.9 Statistical analysis

Results were analyzed using GraphPad Prism™ (version 6.0 for Windows), and one-way analysis of variance (ANOVA) and Student’s t-test were used for comparisons among groups. Differences were considered significant at *P* < 0.05. The experiment was performed twice, and similar results were obtained. The data shown are representative of one whole experiment.

## 3 Results

### 3.1 Blue LED phototherapy presents *in vitro* antileishmanial activity against distinct parasite species

**Figure 1:**
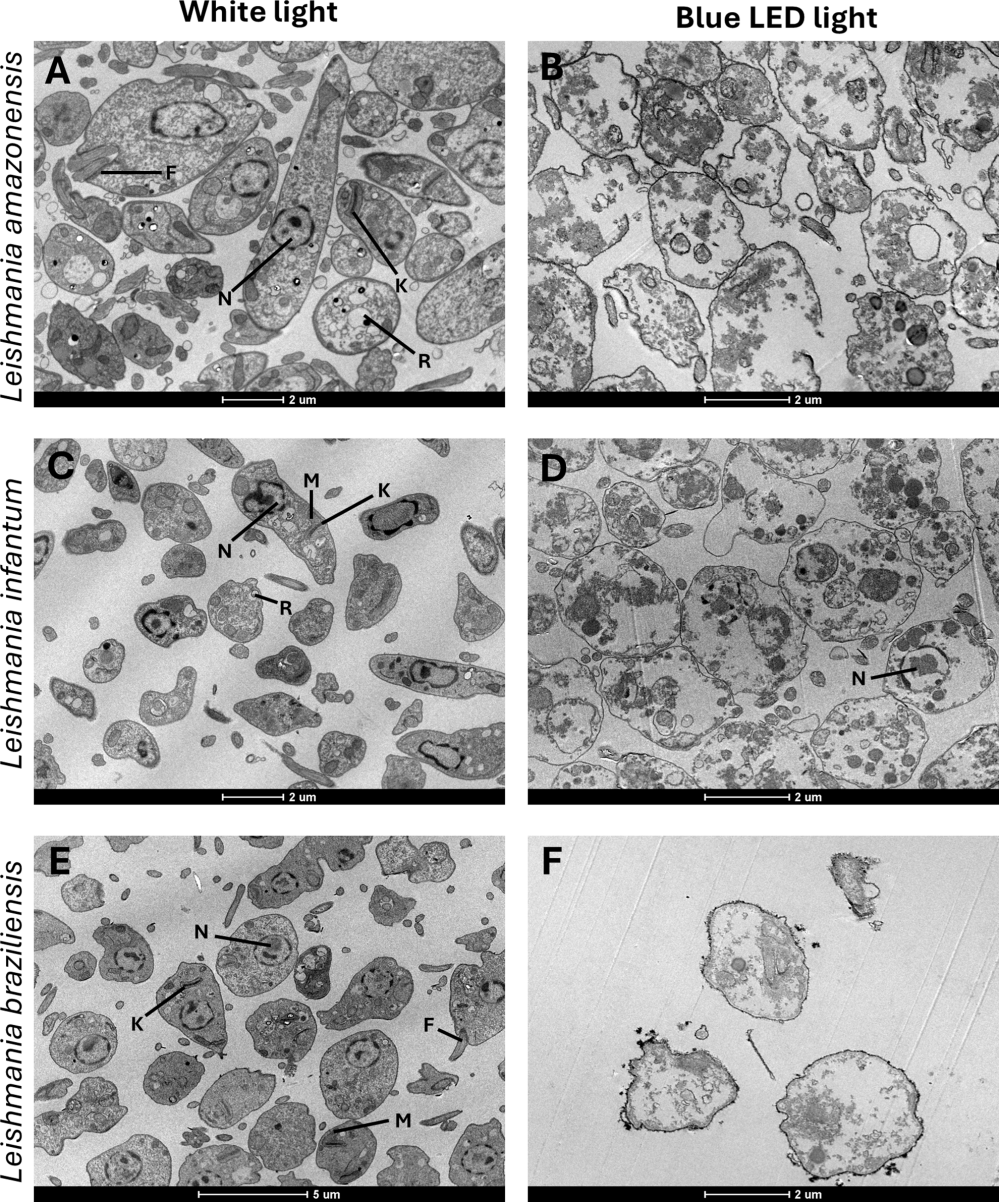
Ultrastructural comparison of *Leishmania* under white light and blue LED phototherapy conditions. The left column shows representative ultrastructural images of promastigotes of *Leishmania amazonensis* (IFLA/BR/1967/PH-8) **(A)**, *L. braziliensis* (MHOM/BR/1975/M2904) **(C)**, and *L. infantum* (MHOM/BR/1970/BH46) **(E)** cultured in Schneider’s medium at 24°C without treatment, under white light exposure. The right column depicts promastigotes of *L. amazonensis* **(B)**, *L. braziliensis* **(D)**, and *L. infantum* **(F)** following daily exposure to blue LED phototherapy (12 hours/day) for 5 days. Structural features include the flagellum (F), mitochondrion (M), reservosome (R), nucleus (N), and kinetoplast (K). Scale bars denote magnification (2um).

We evaluated the *in vitro* activity of blue LED phototherapy against distinct *Leishmania* species, *i.e*., *L. amazonensis, L. infantum* and *L. braziliensis*, which received the treatment for 8 hours per day for 5 consecutive days. Controls were treated with AmpB. Results showed that exposure to natural white light (NL) for five days led to an increase in *Leishmania* numbers across all tested species. In contrast, blue LED phototherapy induced a peak in parasite replication on the third day, followed by a significant proportional reduction on the fourth and fifth days, as well as one day after the end of treatment. Interestingly, the antileishmanial efficacy of amphotericin B (AmpB) was more pronounced against *L. amazonensis* and *L. braziliensis*, but it also effectively reduced *L. infantum* numbers within the first three days of treatment in culture. However, when comparing chemotherapy and phototherapy over five days and one day post-treatment, blue LED phototherapy showed superior leishmanicidal effects (**Table 1**). Scanning electron micrographs of parasites revealed that exposure to white (natural) light for 5 days did not induce ultrastructural changes in *L. amazonensis* (**Figure 1A**), *L. infantum* (**Figure 1C**), or *L. braziliensis* (**Figure 1E**). In contrast, blue LED light therapy caused alterations in the surface membrane structure and disruption of intracellular organelles in all three species: *L. amazonensis* (**Figure 1B**), *L. infantum* (**Figure 1D**), and *L. braziliensis* (**Figure 1F**). Blue LED treatment applied for 24h in infected macrophages reduced the infection percentage by 42.4%, 66.8%, and 42.5% when *L. amazonensis, L. infantum* and *L. braziliensis* promastigotes were used, respectively, and by 50.6%, 80.7%, and 71.8%, respectively, after 48h of treatment (**Table 2**). Using AmpB, reductions were 34.6%, 71.8%, and 53.0%, respectively, after 24h of treatment; and 54.8%, 87.5%, and 73.5%, respectively, after 48h of treatment. Regarding the reduction in the number of recovered amastigotes, values were 47.0%, 75.6%, and 44.3%, respectively, when blue LED phototherapy was used for 24h in *L. amazonensis, L. infantum* and *L. braziliensis*, respectively; and 76.8%, 86.8%, and 89.5%, when treatment was performed by 48h, respectively. Using AmpB, reductions were 35.8%, 78.0%, and 76.0%, after 24h of treatment; and 78.8%, 93.4%, and 89.7%, when treatment was performed by 48h, respectively (**Table 2**).

**Table 1:** *In vitro* antileishmanial activity after treatment with blue LED phototherapy.

	*Leishmania* spp. (parasite concentration x 10^6^ per mL)								
Treatment (day)	*L. amazonensis*			*L. infantum*			*L. braziliensis*		
	bLEDp	AmpB	NL	bLEDp	AmpB	NL	bLEDp	AmpB	NL
1^st^	1.00	1.00	1.00	1.00	1.00	1.00	1.00	1.00	1.00
2^nd^	4.75	0.12	6.00	6.25	3.75	23.80	11.30	0.25	22.50
3^rd^	8.75	0.14	32.50	11.30	12.50	38.80	17.50	3.38	33.80
4^th^	1.50	0.75	52.50	7.50	21.30	47.50	8.13	11.00	65.00
5^th^	0.75	17.80	75.00	8.75	37.50	88.80	9.63	50.00	86.30
One day after	0.50	72.50	67.50	6.25	33.80	85.00	8.00	42.75	93.80

*L. amazonensis*, *L. infantum* and *L. braziliensis* stationary promastigotes were treated with blue LED phototherapy for 8h/day of exposure in 5 consecutive days. Amphotericin B (0.1 μg/mL) was used as a drug control. Daily counts of the parasites were performed by diluting well aliquots at 10 or 100 times, according to the parasite viability. Three independent experiments were performed, and results were similar. bLEDp, blue LED phototherapy; AmpB, amphotericin B; NL, natural light.

**Table 2:** Treatment of infected macrophages using blue LED phototherapy.

		Infection before treatment (%)			Infection after treatment (%)			Reduction of infection (%)			Reduction of amastigote number (%)		
		*L. amaz.*	*L. inf.*	*L. braz.*	*L. amaz.*	*L. inf.*	*L. braz.*	*L. amaz.*	*L. inf.*	*L. braz.*	*L. amaz.*	*L. inf.*	*L. braz.*
24 h	Infected Mø bLEDp	74.47	55.64	71.66	42.92	18.46	41.20	42.36	66.83	42.50	46.95	75.64	44.32
	AmpB	74.47	55.64	71.66	48.74	15.66	33.65	34.55	71.85	53.04	35.82	77.98	76.02
48 h	Infected Mø bLEDp	87.32	62.44	83.95	43.12	12.03	23.69	50.61	80.73	71.78	76.82	86.81	89.45
	AmpB	87.32	62.44	83.95	39.45	7.84	22.25	54.82	87.45	73.50	78.77	93.35	89.71

Murine macrophages were infected with *L. amazonensis*, *L. infantum* or *L. braziliensis* promastigotes (at a ratio of 10 parasites per macrophage) and treated with blue LED phototherapy or amphotericin B (1.0 μg/mL), for 24 or 48 h at 37°C, in both cases. After, cells were stained using the panoptic method. The percentage of infected macrophages and the number of recovered amastigotes were determined by counting 200 cells in triplicate through an optical microscope. Three independent experiments were performed, and the results were similar. bLEDp, blue LED phototherapy; AmpB, amphotericin B; Mø, macrophages.

### 3.2 Blue LED phototherapy induces the development of a Th1-type immune response in treated and *L. amazonensis*-infected mice

**Figure 2:**
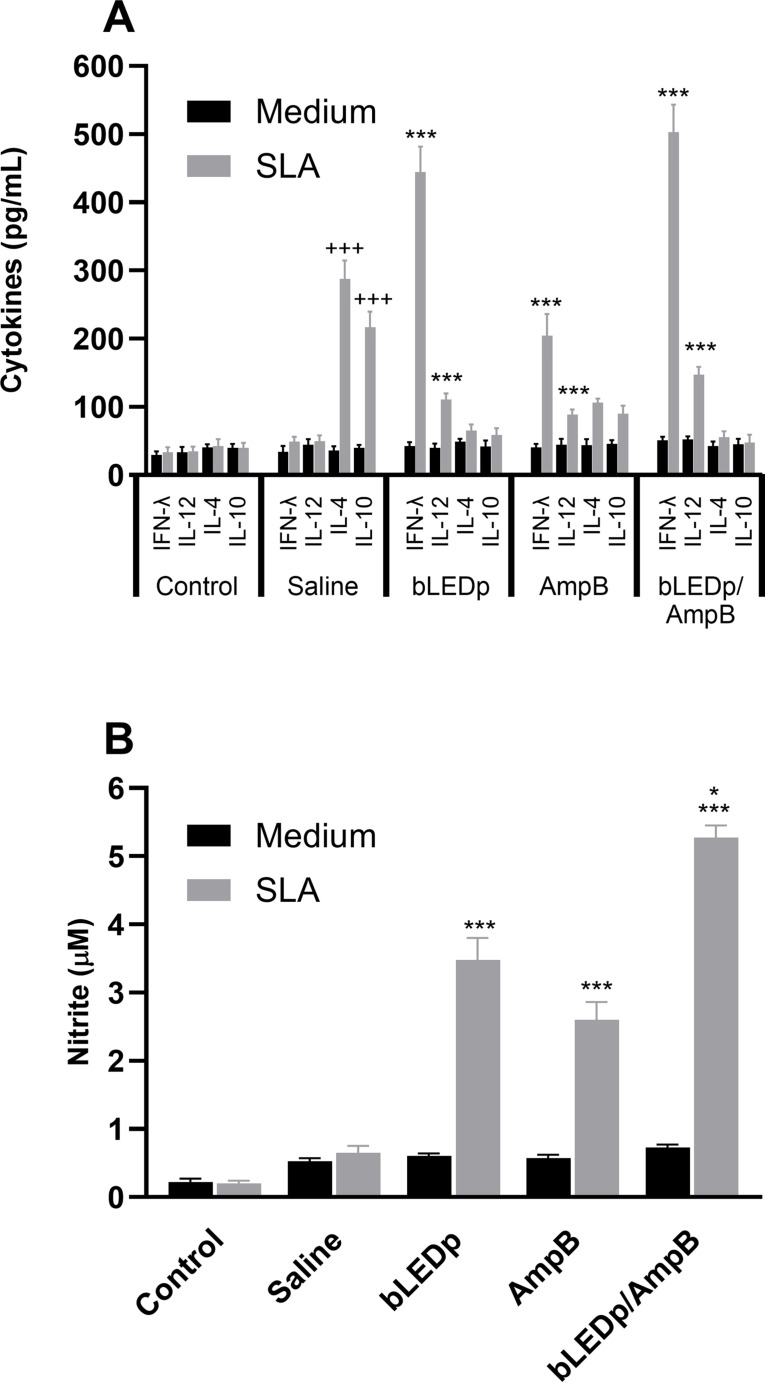
Cytokine production and nitrite secretion after treatment with blue LED phototherapy. Mice were infected with *L. amazonensis* promastigotes and treated with amphotericin B (AmpB), blue LED phototherapy (bLEDp) or associated with AmpB (bLEDp/AmpB), or they received only saline. Then, one day after treatment, animals were euthanized, and spleen cells were cultured in RPMI medium and stimulated with soluble *Leishmania* antigen (SLA) for 48 h at 37°C at 5% CO_2_. Levels of IFN-γ, IL-4, IL-10 and IL-12p70 cytokines were measured in culture supernatant through a capture ELISA, and results are shown **(A)**. The same cell supernatant was used to measure the levels of antileishmanial nitrite, and results are also shown **(B)**. Two independent experiments were performed, and bars indicate the mean ± standard deviation of the groups. (^*^) indicates a statistically significant difference between the bLEDp and AmpB groups (*P* < 0.05). (^***^) indicates a statistically significant difference between the control and saline groups (*P* < 0.001). (^+++^) indicates a statistically significant difference among bLEDp, AmpB and bLEDp/AmpB groups (*P* < 0.05).

**Figure 3:**
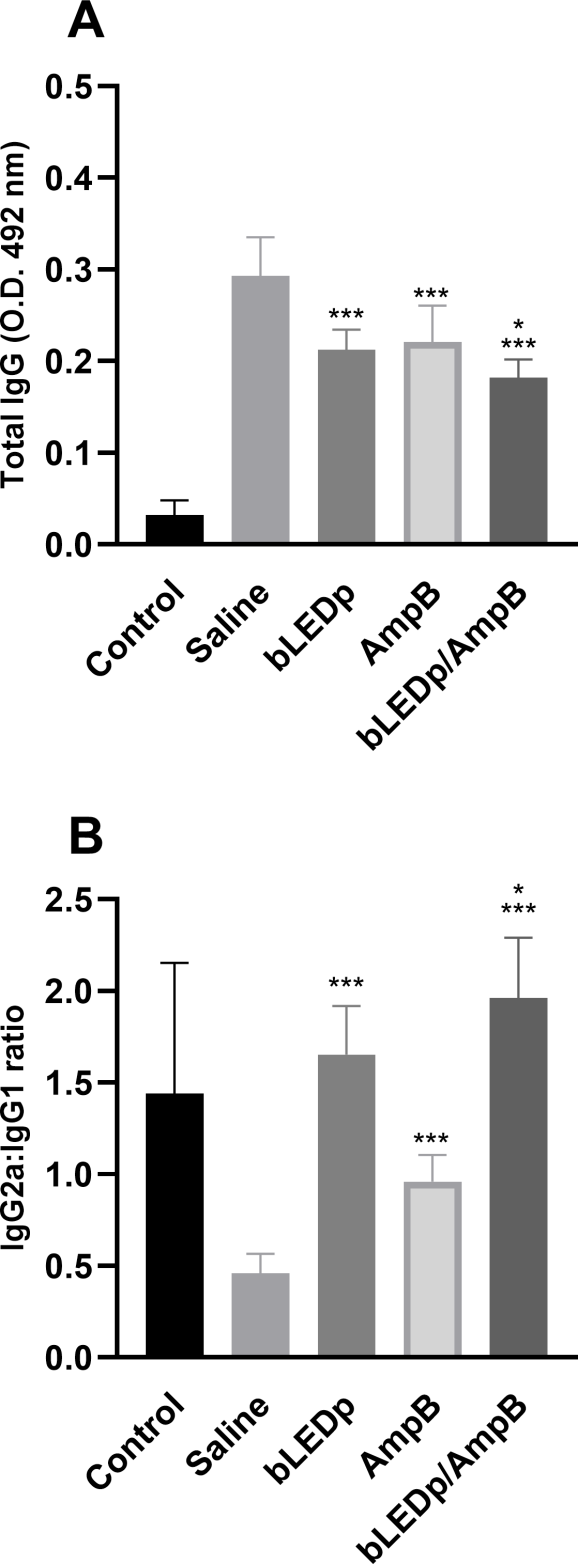
Antibody production after treatment with blue LED phototherapy. Mice were infected with *L. amazonensis* promastigotes and treated with saline, amphotericin B (AmpB), blue LED phototherapy (bLEDp) or in association with AmpB (bLEDp/AmpB. One day after treatment, sera samples were collected and used to evaluate the production of anti-soluble *Leishmania* antigen (SLA) IgG total **(A)** and the IgG2a/IgG1 ratio **(B)** antibodies, through an indirect ELISA. Two independent experiments were performed, and bars indicate the mean ± standard deviation of the groups. (^*^) indicates a statistically significant difference in the bLEDp and AmpB groups (*P* < 0.05). (^***^) indicates a statistically significant difference in the control and saline groups (*P* < 0.001).

We evaluated the therapeutic efficacy induced by blue LED phototherapy in *L. amazonensis*-infected mice. To do so, levels of Th1-and Th2-type cytokines were measured in the supernatant of SLA-stimulated spleen cell cultures, and results showed that treatment with blue LED induced the production of higher levels of IFN-γ and IL-12, which were associated with lower IL-4 and IL-10 levels (**Figure 2A**). A Th1-type response was found in mice treated with an association of blue LED and AmpB, when compared to values obtained in the other groups. To evaluate the parasite-specific activation of macrophages in the treated and infected animals, the nitrite secretion was investigated in the culture supernatant, and results corroborated those described for the cytokine levels, since the treatment with blue LED plus AmpB induced higher levels of antileishmanial nitrite compared to the others (**Figure 2B**). We also evaluated antibody production after treatment (**Figure 3**) and, mice receiving AmpB, blue LED and, mainly, blue LED plus AmpB produced higher levels of anti-SLA IgG2a antibodies compared to IgG1 levels. Otherwise, infected and untreated mice (saline group) produced higher levels of IgG1 antibodies, in comparison to IgG2a levels (**Figure 3**).

### 3.3 Blue LED phototherapy induces a reduction in the *L. amazonensis* burden after treatment of infected mice

**Figure 4:**
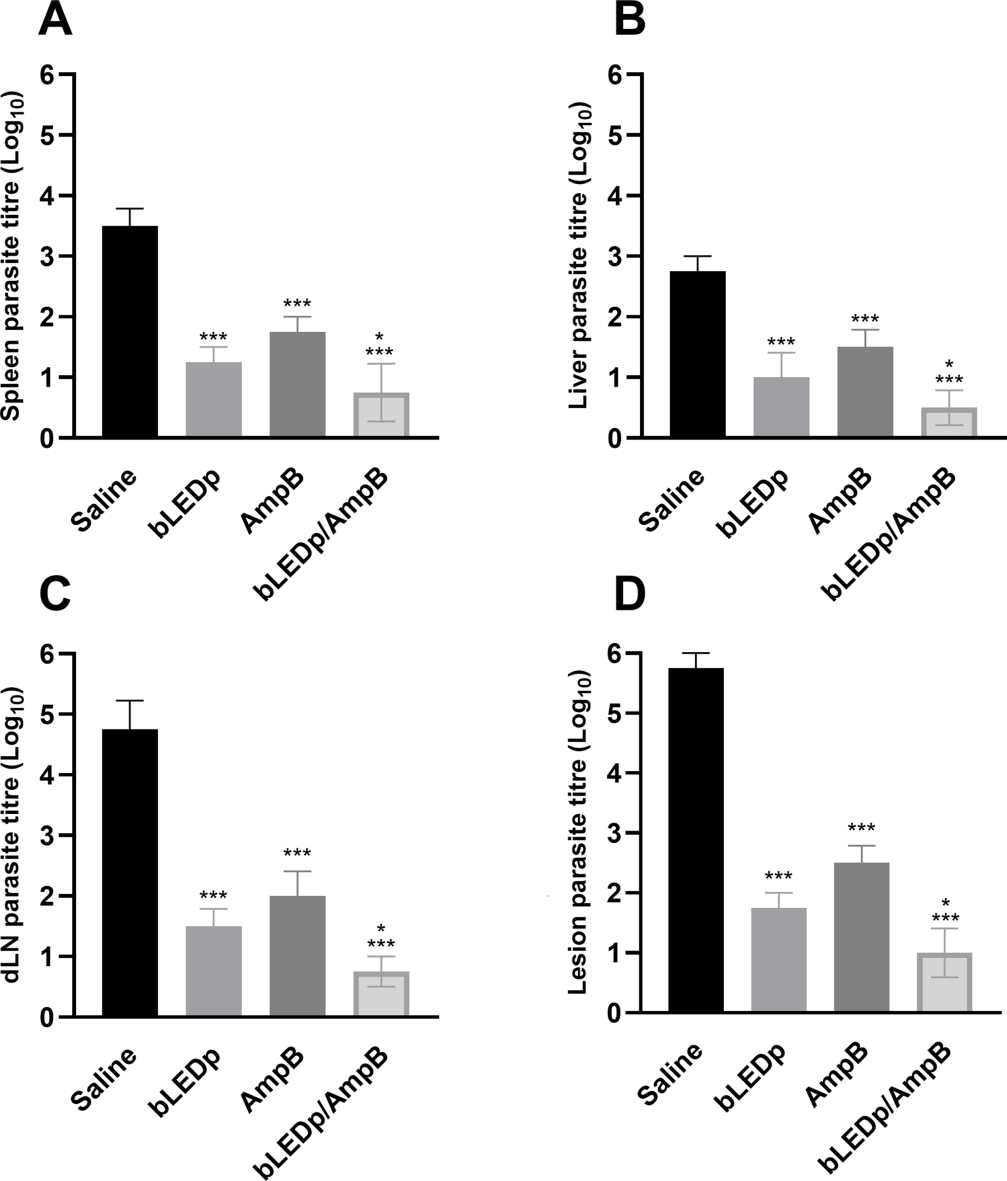
*Leishmania amazonensis* burden evaluated after blue LED phototherapy by a limiting dilution technique. *L. amazonensis*-infected mice were treated with amphotericin B (AmpB), blue LED phototherapy (bLEDp) alone or associated with AmpB (bLEDp/AmpB). Other animals were infected and received only saline. Three days after treatment, animals were euthanized and spleen **(A)**, liver **(B)**, draining lymph node (dLN) **(C)**, and skin lesion **(D)** were collected to evaluate the parasite load, through a limiting dilution technique **(A)**. In addition, their skin lesions were used in a qPCR assay to estimate the parasite load, with results being normalized by number of parasites per total DNA. Two independent experiments were performed, and bars indicate the mean ± standard deviation of the groups. (^*^) indicates a statistically significant difference between bLEDp and AmpB groups (*P* < 0.05). (^***^) indicates a statistically significant difference in the control and saline groups (*P* < 0.001).

**Figure 5:**
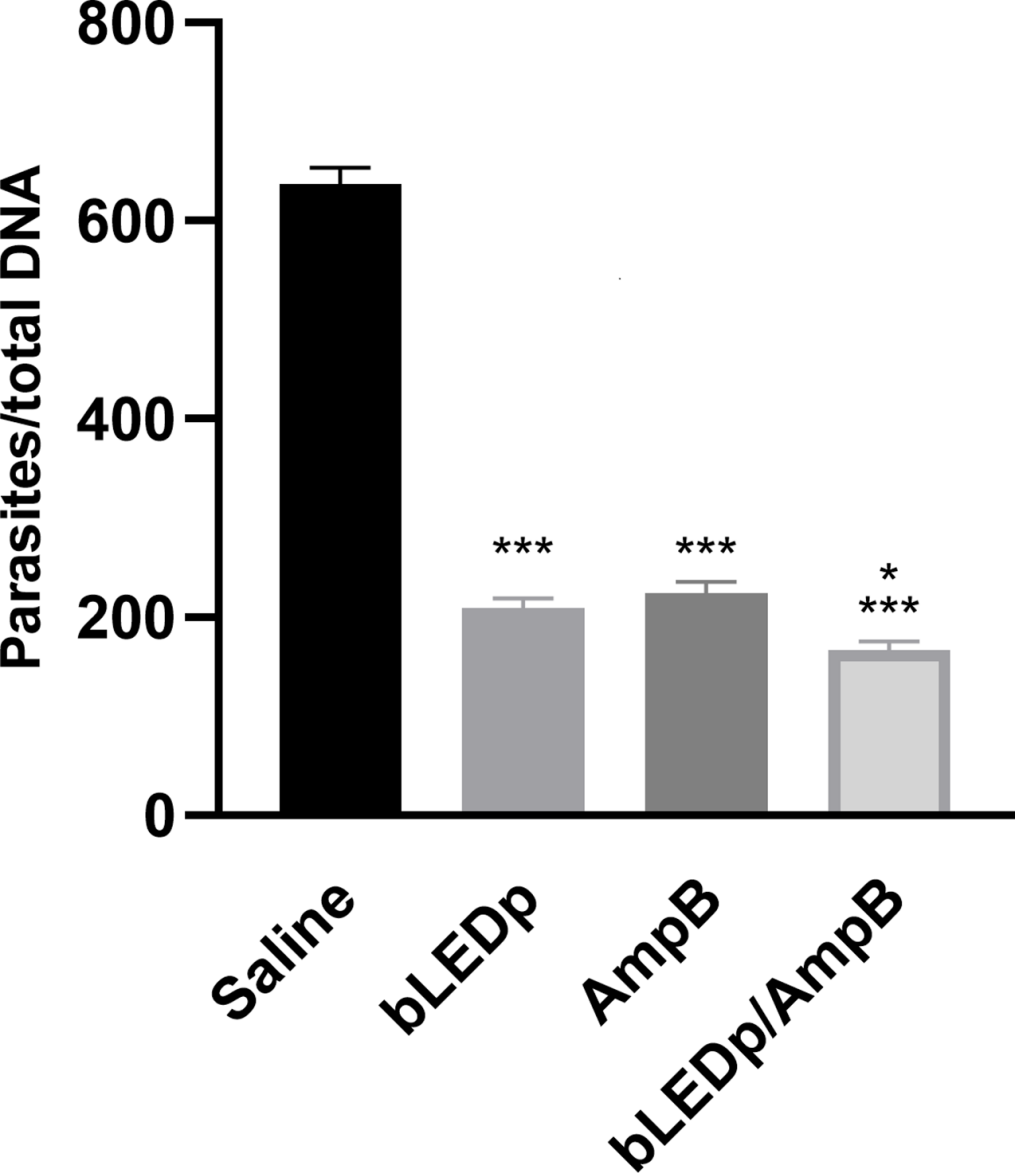
Skin lesion parasitism estimated through a qPCR assay. *L. amazonensis*-infected mice received saline or were treated with blue LED phototherapy (bLEDp) alone, with amphotericin B (AmpB), or with association of both treatments (bLEDp/AmpB). One day after treatment, animals were euthanized, and skin lesions were collected, macerated, and used to estimate the parasite load through a qPCR technique. Results were normalized by number of parasites per total DNA. Two independent experiments were performed, and bars indicate the mean plus standard deviation of the groups. (^*^) indicates a statistically significant difference in the bLEDp and AmpB groups (*P* < 0.05). (^***^) indicates a statistically significant difference in the control and saline groups (*P* < 0.001).

The parasite burden was evaluated in livers, spleens, dLNs and skin lesions of treated and infected animals, and results showed significant reductions in parasitism in mice receiving blue LED alone or in combination with AmpB, compared to values found in the saline group mice, with reductions of 4.0- and 4.8-log, respectively, in their lesions; 1.8- and 2.3-log, respectively, in their livers; 2.3- and 2.7-log, respectively, in their spleens; and 3.2- and 4.0-log, respectively, in their dLNs (**Figure 4**). A qPCR assay was also performed on the skin lesions, and results showed that mice receiving blue LED associated with AmpB presented greater reductions in parasite load, of 67.0% and 74.0%, respectively, compared to data described for the saline group (**Figure 5**).

## 4 Discussion

Treatment for leishmaniasis faces significant challenges, including drug toxicity, high costs, and the emergence of drug-resistant strains (28). In this context, the discovery of new antileishmanial targets and strategies is urgently needed (7). In the present study, we explored blue LED phototherapy as a novel antileishmanial approach through *in vitro* and *in vivo* experiments. The therapy proved effective in reducing parasite viability in *in vitro* cultures and decreasing parasite burden in infected macrophages. Additionally, *in vivo* experiments, blue LED phototherapy helped *Leishmania*-infected mice control the infection and develop a therapeutic phenotype, offering protection against the disease. To the best of our knowledge, this is the first study to demonstrate the efficacy of blue LED phototherapy against *Leishmania* spp.

Our initial experiments evaluated the antileishmanial effects of blue LED phototherapy *in vitro* by applying the therapy directly to parasite cultures at a single frequency without photosensitizing molecules. A significant reduction in parasite viability was observed after three days of treatment, suggesting a cumulative effect (29, 30). Similar findings were reported by Ivanova et al. (22), who showed that blue LED light inhibited *T. cruzi* cultures. The exposure of microorganisms - including bacteria, protozoa, and viruses - to distinct light wavelengths has demonstrated efficacy in inactivating these pathogens, supporting their potential for managing infectious diseases (19, 31).

The mechanisms underlying blue LED phototherapy’s effects remain under investigation. One proposed mechanism involves intracellular photosensitizing chromophores, which generate reactive oxygen species (ROS), leading to pathogen death (32, 33). Another mechanism suggests disruption of lipid and protein structures, impairing intracellular transport and organelle organization (20, 34). Additionally, blue LED light may modulate the host’s immune response, further contributing to its therapeutic effects (35, 36). Notably, the biological effects of blue LED phototherapy can vary across microorganisms due to differences in environmental conditions, light wavelength, and exposure duration (37, 38).

Following promising *in vitro* results, we selected *L. amazonensis* for the *in vivo* experiments, as it is a well-established model for cutaneous leishmaniasis (CL) and generates visible lesions at the site of infection, which is crucial for evaluating phototherapy’s effects. The ability to directly target the lesions with blue LED light is an advantage when studying therapeutic strategies. While *L. braziliensis* also causes cutaneous leishmaniasis, we opted to focus on *L. amazonensis* for the *in vivo* experiments due to the limitations of the BALB/c mouse model. BALB/c mice are not typically susceptible to *L. braziliensis*, as they do not develop significant lesions and tend to spontaneously resolve the infection, which would not allow for meaningful evaluation of phototherapy’s impact (39, 40).

Leishmania infections in mammals are often associated with the development of a Th2-type immune response. This response is characterized by the production of cytokines such as IL-4, IL-5, IL-6, IL-10, and IL-13, which deactivate infected macrophages and allow parasite dissemination at infection sites and internal organs (41, 42). The Th2 cytokine environment promotes the recruitment of anti-inflammatory cells, facilitating parasite persistence. In contrast, a Th1-type response - mediated by cytokines such as IFN-γ, IL-2, GM-CSF, and IL-12 - induces inflammation and enhances parasite elimination (43, 44).

In our study, blue LED phototherapy increased levels of pro-inflammatory cytokines IFN-γ and IL-12, which synergized with nitric oxide production to eliminate intracellular parasites (27, 45). In contrast, control group mice exhibited elevated levels of IL-4 and IL-10, which suppressed macrophage activation and promoted disease progression (46). The immunostimulatory effects of blue LED phototherapy likely contributed to the reduced parasite burden in treated animals, as evidenced by lower IL-10 levels and increased IFN-γ and IL-12 production. This suggests that blue LED phototherapy not only exerts direct antileishmanial effects but also modulates the host immune response, offering a dual mechanism for controlling the infection.

Distinct *Leishmania* species are responsible for various clinical manifestations of leishmaniasis. For example, species such as *L. major*, *L. braziliensis*, *L. amazonensis*, and *L. tropica* cause cutaneous leishmaniasis (CL), while *L. donovani* and *L. infantum* are associated with visceral leishmaniasis (VL) (47). In our study, we verified that blue LED phototherapy exhibited antileishmanial activity against three *Leishmania* species, significantly reducing cell viability *in vitro* experiments. The leishmanicidal effects of blue LED phototherapy were partially attributed to the disruption of intracellular organelles and cell membranes. This disruption impairs the parasites’ ability to regulate their metabolic environment, ultimately leading to cell death.

Although the current study did not investigate the precise mechanisms underlying these structural alterations, previous research on *Staphylococcus aureus* offers valuable insights. Blue light phototherapy has been shown to induce changes in membrane potential and stimulate free radical production via photo-acceptor molecules in bacteria (48). These findings may serve as a foundation for further exploration of similar mechanisms in *Leishmania*.

Currently, drugs such as pentavalent antimonials, miltefosine, pentamidine, and AmpB are commonly used to treat leishmaniasis; however, these treatments often cause significant toxic effects in patients (49). In our study, combining AmpB with blue LED phototherapy enhanced antileishmanial activity compared to AmpB alone. Similar synergistic effects have been reported in other studies, where AmpB was combined with immunogenic antigens in immunotherapeutic strategies tested in *Leishmania*-infected mice (26, 50, 51). Future studies may explore the potential of combining blue LED phototherapy with immunogenic candidates as part of such immunotherapeutic protocols.

To the best of our knowledge, this is the first study to demonstrate that blue LED phototherapy can control *in vitro* infections of *Leishmania* cultures following daily exposure. Additionally, our results indicate that this strategy may contribute to modulating the immune response in infected mice and reducing parasite burden in tissues and internal organs. While further research is needed to confirm the *in vivo* antileishmanial effects of blue LED phototherapy, the findings presented here provide proof-of-concept evidence supporting its potential use for the treatment of TL.

## Data Availability

The raw data supporting the conclusions of this article will be made available by the authors, without undue reservation.
